# Prognostic Value of Neutrophil-Related Factors in Locally Advanced Cervical Squamous Cell Carcinoma Patients Treated with Cisplatin-Based Concurrent Chemoradiotherapy

**DOI:** 10.1155/2016/3740794

**Published:** 2016-03-20

**Authors:** Yan-Yang Wang, Zhou-Lan Bai, Jian-Li He, Yan Yang, Ren Zhao, Ping Hai, Hong Zhe

**Affiliations:** ^1^Department of Radiation Oncology, General Hospital of Ningxia Medical University, Yinchuan, Ningxia 750004, China; ^2^Cancer Institute, Ningxia Medical University, Yinchuan, Ningxia 750004, China

## Abstract

The aim of this study was to explore the relationship between neutrophil-related factors, including neutrophil-lymphocyte ratio (NLR) and the responses of neutrophil to granulocyte colony-stimulating factors (RNG), and the prognosis of patients with locally advanced cervical squamous cell carcinoma (LACSCC) undergoing cisplatin-based concurrent chemoradiotherapy (CCCRT). A total of sixty LACSCC patients were enrolled in this study. We analyzed the association of NLR or RNG with clinicopathologic characteristics of these patients. The prognostic factors were evaluated by univariate and multivariate survival analysis. The optimal cut-off value of the NLR was determined to be 2.0 for the overall survival (OS). A higher level of the NLR was associated with younger age (*P* = 0.017) and higher baseline platelet count (*P* = 0.040). NLR was identified to be the only independent prognostic factor for OS by multivariate analysis (*P* = 0.037). The median RNG was 3.01, with a range of 1.19–16.84. RNG level was significantly associated with lymph node metastasis of these patients (*P* = 0.023). And higher RNG was identified as being a closely independent poor prognostic factor for OS (*P* = 0.055). This study showed that NLR and RNG may be used as potential biomarkers for survival prediction in patients with LACSCC receiving CCCRT.

## 1. Introduction

Cervical cancer is the second most common type of cancer and the leading cause of cancer death in female in developing countries [1]. In patients with advanced stage disease, the standard treatment is cisplatin-based concurrent chemoradiotherapy (CCCRT), followed by brachytherapy [2]. Tumor size, lymph node status, International Federation of Gynecology and Obstetrics (FIGO) stage, and pretreatment hemoglobin level were reported to be independent prognostic factors for locally advanced cervical cancer [3, 4]. However, to further improve the treatment outcome of these patients, more prognostic factors are still needed.

Recently, neutrophil-lymphocyte ratio (NLR) was evaluated as a prognostic indicator in many types of cancer including gastrointestinal tract malignancies [5], hepatocellular carcinoma [6], pancreatic cancer [7], and non-small-cell lung cancer [8]. Although the prognostic significance of NLR has also been investigated in cervical cancer [9–12], the value of NLR in survival prediction of patients with locally advanced cervical squamous cell carcinoma (LACSCC) who received CCCRT remains unknown.

Neutropenia is the most common therapy related toxicity of LACSCC patients who received CCCRT [13, 14]. The duration of neutropenia can be minimized with the use of granulocyte colony-stimulating factors (G-CSFs) [15]. However, the responses of neutrophil to G-CSFs (RNG) among patients are variable [16–18], which may impact the prognosis of LACSCC. To the best of our knowledge, the prognostic value of RNG in LACSCC has never been investigated.

In current study, we hypothesized that neutrophil-related factors, including NLR and RNG, were prognostic indicators of patients with LACSCC who underwent CCCRT. The prognostic values of NLR and RNG in LACSCC were evaluated.

## 2. Materials and Methods

### 2.1. Patient Population

The study included 60 consecutive patients with pathologically confirmed cervical cancer who underwent CCCRT from June 2009 to June 2010 at General Hospital of Ningxia Medical University. Clinicopathologic information of these patients, including age, pathologic diagnosis, histologic grade, tumor size, lymph node status, parametrial invasion, FIGO stage, baseline hemoglobin level, and platelet count, was obtained from medical records. Patients with hematologic, autoimmune, or infectious diseases were excluded. This study was approved by the ethics committee of our hospital.

### 2.2. Treatment and Follow-Up

The pretreatment evaluation included a review of the patient's history, physical examination, performance status, gynecologic examination, chest X-ray, complete blood count, blood chemistry, and abdominal-pelvic magnetic resonance imaging (MRI). Cystoscopy and sigmoidoscopy were performed when indicated. Radiotherapy included external beam radiotherapy up to 50 Gy and low-dose rate brachytherapy, six applications of 6 Gy. Chemotherapy consisted of weekly intravenous cisplatin administration (40 mg/m^2^) for 5 cycles concomitant with external pelvic radiation. Treatment response was clinically assessed according to RECIST version 1.1 [19]. Treatment toxicity was classified according to the National Cancer Institute Common Terminology Criteria for Adverse Events (CTCAE; version 4.0) [20].

The patients were followed up every three months for the first two years, in six-month intervals for the next three years, and every year thereafter. During the routine follow-up, imaging studies including CT or MRI and chest X-ray were performed annually and when tumor recurrence was suspected based on clinical findings or imaging studies, biopsy of that lesion was performed on a case-by-case basis. Overall survival (OS) time was defined as the interval between date of the completion of treatment and death, or the last follow-up, and progression-free survival (PFS) time was defined as the period from date of the completion of treatment to the occurrence of local recurrence or distant metastasis or the last follow-up. Patient follow-up was maintained until death or the cut-off date of June 2015.

### 2.3. Definition of NLR and RNG

All baseline white cells and differential counts were obtained within one week before CCCRT. The NLR was defined as the absolute neutrophil count divided by the absolute lymphocyte count. During the CCCRT, some patients may develop neutropenia. The absolute neutrophil count of first neutropenia during treatment was defined as *N*_1_. After the G-CSFs therapy, the absolute neutrophil count was defined as *N*_2_. RNG was calculated as *N*_2_/*N*_1_.

### 2.4. Statistical Analysis

The associations between NLR or RNG and the clinicopathologic variables were analyzed by a chi-square test. The overall and progression-free survival curves were calculated according to the Kaplan-Meier method with the log-rank test. Prognostic factors with significance values of *P* less than 0.05 in a univariate analysis were entered into a multivariate analysis, which was conducted using the Cox proportional hazards model with the backward likelihood method. All reported *P* values were two-sided, and *P* less than 0.05 was considered statistically significant. SPSS 13.0 (SPSS Inc., Chicago, IL) was used for the statistical analysis.

## 3. Results

### 3.1. Patient Characteristics and Treatment Outcome

The enrolled 60 LACSCC patients had a median age of 53 years (range 36 to 80 years). Histologically, all primary tumors were squamous cell carcinoma. Staging was performed according to FIGO staging system classification. Clinicopathologic characteristics of these patients are listed in Table 1. All patients received the external beam radiotherapy and brachytherapy as indicated in the protocol. For myelosuppression and infectious complications, only 34 (56.67%) patients received 5 cycles of cisplatin-based concurrent chemotherapy. According to RECIST, the complete response rate of the whole cohort is 56.7%. The median follow-up time of the censored patients was 58 (range, 7–70) months. During the follow-up period, 23 patients were recurrent, and 19 patients were dead. The 5-year PFS and OS of the whole cohort were 56.60% and 61.30%, respectively.

### 3.2. The Prognostic Value of NLR

The NLR value of the whole study population ranged from 0.99 to 6.91 with a median of 2.40. The optimal cut-off value of the NLR was determined to be 2.0 for the OS. We analyzed the association of the different NLR levels with clinicopathologic characteristics of patients. There were no significant differences in the clinicopathologic characteristics between the patients with high NLR level and those with low level, except age and baseline platelet count. It was found that a higher NLR level (≥2.0) was associated with younger age (*P* = 0.017) and higher baseline platelet count (*P* = 0.040). However, the NLR was not significantly associated with the response of cervical cancer to CCCRT (Table 1).

**Figure 1:**
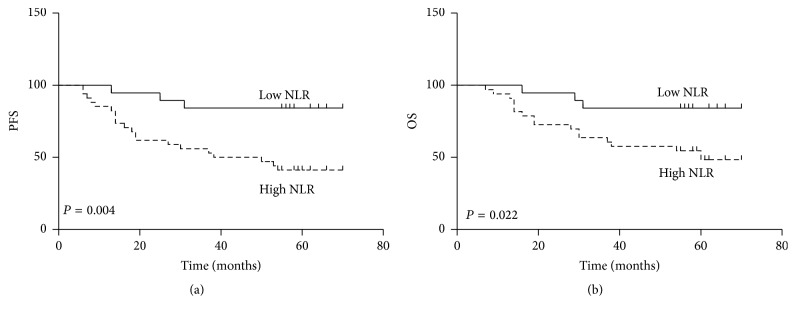
Kaplan-Meier survival curves for progression-free survival (PFS) (a) and overall survival (OS) (b) of LACSCC patients with a high NLR and those with a low NLR.

Compared with a lower NLR, a higher NLR was associated with significant worse PFS (*P* = 0.004) and OS (*P* = 0.022) (Figure 1). Other significant prognostic indicators identified by univariate analysis included lymph node metastasis (*P* = 0.029) and FIGO stage (*P* = 0.044) for OS. These variables were selected for multivariate analysis using a backward likelihood method, and only NLR (*P* = 0.037) was identified to be independent prognostic factor for OS. For PFS, only lymph node status (*P* = 0.032) was identified as an independent prognostic factor in univariate analysis. The detailed results were shown in Tables 2 and 3.

### 3.3. The Prognostic Value of RNG

In the whole cohort, 35 (58.33%) patients experienced neutropenia, including 19 Grade 1, 14 Grade 2, and 2 Grade 3 cases. RNG value of this subgroup patients ranged from 1.19 to 16.84 (median, 3.01). The median value 3.01 was selected as the cut-off of high and low RNG levels. The complete rates of 5 cycles concurrent chemotherapy in high and low RNG group were 55.56% and 58.82% (*P* = 0.832), respectively. The mean amounts of G-CSFs administration in high and low RNG group were 583.34 ± 69.66 *μ*g and 566.71 ± 85.58 *μ*g (*P* = 0.960), respectively.  Table 4 shows the relationship of different levels of RNG and the clinicopathologic characteristics of patients. The RNG level was significantly associated with lymph node status of these patients (*P* = 0.023).

**Figure 2:**
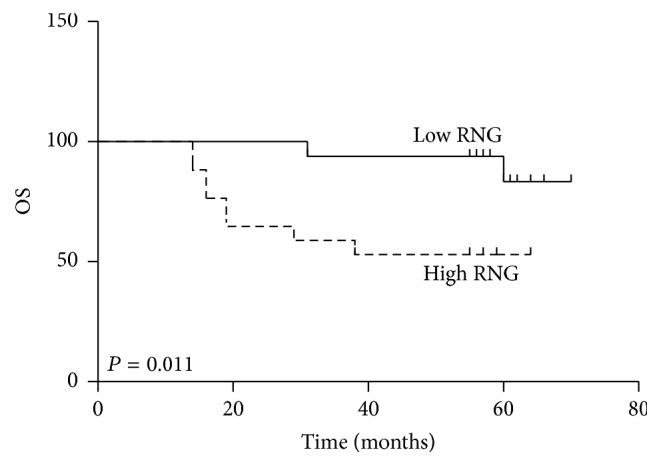
Kaplan-Meier survival curves for overall survival (OS) of LACSCC patients with a high RNG and those with a low RNG.

According to the Kaplan-Meier analysis, the OS was significantly shorter for the patients in the high RNG group than for their low counterparts (52.94% versus 83.33%, *P* = 0.011) (Figure 2). However, the difference of PFS between these two groups did not meet statistical significance. We performed univariate analyses to determine the prognostic factors for OS of these patients. Lymph node status (*P* = 0.025) and RNG level (*P* = 0.002) were prognostic predictors for poor OS. The multivariate analysis confirmed that RNG level was a closely independent prognostic predictor for OS (*P* = 0.055). The detailed results were shown in Tables 5 and 6.

## 4. Discussion

It has been recognized that inflammation is an important regulator in the genesis, progression, and metastasis of malignant diseases [21]. Patient response to malignant tumors comprises not only changes in the tumor microenvironment, but also systemic inflammatory alternations [22]. More recently, NLR was defined as a potential marker to determine inflammation in various malignant diseases [5–8]. For cervical cancer, the association between NLR and clinicopathologic characteristics of patients, including age, tumor size, lymph node metastasis, FIGO stage, and depth of stromal infiltration, has been demonstrated. The correlation between the increase of NLR and prognosis of cervical cancer has also been found in these studies [9–12]. However, there was large heterogeneity in the disease stage and treatment modality of these studies; thus the prognostic value of NLR in cervical cancer, especially for LACSCC, remains controversial.

In this study, we assessed the clinicopathologic relevance and prognostic value of NLR in LACSCC patients who underwent CCCRT. We found that NLR was associated with some important clinicopathologic characteristics of LACSCC, including patient age and baseline platelet count. In addition, Kaplan-Meier analysis confirmed that patients with high NLR had a significantly poorer PFS and OS compared with patients with low NLR. Cox regression analysis revealed that NLR was an independent prognostic indicator for OS of patients with LACSCC, but not for PFS. These findings were also confirmed in other studies (Table 7). Lee et al. [9] reported that patients with cervical cancer of the higher NLR group were younger in age and had more advanced staged disease when compared with those of the lower NLR group. In multivariable analysis, higher pretreatment NLR was identified as being an independent poor prognostic factor for survival. Zhang et al. [11] evaluate the clinicopathologic and prognostic values of NLR in patients with cervical cancer undergoing primary radical hysterectomy with pelvic lymphadenectomy. They found NLR was highly associated with depth of stromal infiltration and lymph node metastasis. Multivariable analysis showed that the NLR was an independent prognostic marker for PFS, but not for OS. They concluded that the preoperative NLR may be used as a potential and easy biomarker for survival prognosis in patients with cervical cancer receiving initial radical hysterectomy with pelvic lymphadenectomy. Mizunuma et al. [12] retrospectively analyzed 56 patients with squamous cell carcinoma of the uterine cervix who underwent RT or concurrent chemotherapy and RT. They demonstrated that NLR was a significant prognostic factor for PFS and OS. Patients with a high NLR had significantly shorter PFS and OS than those with a low NLR. Furthermore, in comparison to a high NLR, a low NLR was significantly associated with a complete response. However, we observed no significant correlations between the NLR and response rate of CCCRT in this study. The possible reason for the differences may be attributed to the various stage of the enrolled patients and the different treatment regimens between these two studies.

The specific mechanisms as to the relationship between high NLR and poor survival of cancer have yet to be identified. There are some points that can be used for interpreting these results. Firstly, pretreatment neutrophil and lymphocyte numbers indicate the level of systemic inflammation. Inflammation is known to change the microenvironment of a tumor and promote angiogenesis and metastasis [21, 23]. Secondly, high-density circulating neutrophils may adversely affect the tumor-bearing host, resulting in a negative association between neutrophil density and patient survival [11]. Circulating lymphocyte has been shown to secrete cytokines, which prevent proliferation and metastasis of tumor cells and have an important function in cytotoxicity [24]. NLR can reflect the balance between host inflammatory response and immune response. The imbalance of NLR could lead to a negative association with oncologic outcome. Thirdly, neutrophils have been proved to contain and secrete vascular endothelial growth factor, IL-18, and matrix metalloproteinases, which directly contribute to tumor-related angiogenesis, tumor growth, and metastasis [25, 26].

In the second part of our study, we examined the RNG levels in LACSCC and its correlation with patient prognosis. We found that high RNG level was significantly correlated with shorter OS and RNG level was a closely independent prognostic factor for OS of patients with LACSCC. RNG level was significantly associated with lymph node metastasis of LACSCC which has also been demonstrated. However, the mechanism underlying these results has not been elucidated yet. A possible explanation is that G-CSFs induced the production of myeloid derived suppressor cells (MDSCs), especially in the sensitive patients [27]. These MDSCs could cause rapid progression of cervical cancer, leukocytosis, and treatment resistance. Mabuchi et al. depleted MDSCs, which succeeded in inhibiting the progression of cervical cancer, leukocytosis and enhancing radiosensitivity [28]. Additionally, as the production of G-CSFs, neutrophil may also contribute to the progression of cervical cancer. An increased neutrophil count in the peripheral blood has been suggested to be a significant prognostic factor in patients with a variety of cancers, including metastatic melanoma, renal cell carcinoma, non-small-cell lung cancer, breast cancer, head and neck carcinoma, and sarcoma [29]. Vascular endothelial growth factor, interleukin-18, and matrix metalloproteinases, secreted by circulating neutrophils, contribute to the tumor-related angiogenesis, tumor growth, and metastasis [30–32].

Some limitations of this study should be noticed. Firstly, this study is limited by its retrospective design and single-institution experience. Secondly, the lack of analysis on the correlation of these findings in the bloodstream with the intratumoral infiltrate is a major limitation of this study. Finally, the cut-off value for NLR was 2.0, which was selected based on its prognostic value in our data set. It needs to be verified in a validation cohort.

In conclusion, this study highlights the potential of NLR and RNG as additional prognostic indicators in patients with LASCC who had been treated with CCCRT. However, the results of current study need to be validated in larger prospective studies in near future.

## Figures and Tables

**Table 1:** Association between NLR and clinicopathologic characteristics of LACSCC patients.

Clinicopathologic characteristics	NLR, *n* (%)		*P* value
	≥2.0	<2.0	
Age			
≤50 years	21 (77.7)	6 (22.3)	0.017
>50 years	15 (45.4)	18 (54.6)	
Histologic grade			
Well and moderately differentiated	18 (58.0)	13 (42.0)	0.797
Poorly differentiated	18 (62.0)	11 (38.0)	
Tumor size			
≤4 cm	9 (45.0)	11 (55.0)	0.105
>4 cm	27 (67.5)	13 (32.5)	
Parametrial invasion			
No	16 (55.1)	13 (44.9)	0.595
Unilateral	14 (60.8)	9 (39.2)	
Bilateral	6 (75.0)	2 (25.0)	
Clinical lymph node involvement			
cN0	24 (54.5)	20 (45.5)	0.234
cN1	12 (75.0)	4 (25.0)	
FIGO stage			
II	20 (55.5)	16 (44.5)	0.432
III	16 (66.6)	8 (33.4)	
Hemoglobin levels at diagnosis (g/dL)			
≤113	7 (53.8)	6 (46.2)	0.751
>113	29 (61.7)	18 (38.3)	
Platelets at diagnosis (g/dL)			
≤320	9 (90.0)	1 (10.0)	0.040
>320	27 (54.0)	23 (46.0)	
Response			
CR	20 (58.8)	14 (41.2)	1.000
Non-CR	16 (61.5)	10 (38.5)	

NLR, neutrophil-lymphocyte ratio; LACSCC, locally advanced cervical squamous cell carcinomas; FIGO, International Federation of Gynecology and Obstetrics; CR, complete response.

**Table 2:** Univariate survival analysis of PFS and OS in patients with LACSCC.

Clinicopathologic characteristics	PFS		*P* value	OS		*P* value
	Mean ± SD (months)	95% CI		Mean ± SD (months)	95% CI	
Age						
≤50 years	40.24 ± 4.51	−1.775–19.821	0.100	45.36 ± 4.01	−4.218–15.562	0.255
>50 years	49.26 ± 3.23			51.03 ± 3.04		
Histologic grade						
Well and moderately differentiated	47.72 ± 3.27	−16.953–5.247	0.295	49.71 ± 3.69	−11.964–8.132	0.704
Poorly differentiated	41.87 ± 4.68			47.79 ± 3.31		
Tumor size						
≤4 cm	44.84 ± 3.60	−13.095–9.726	0.769	48.79 ± 3.17	−9.832–11.016	0.910
>4 cm	46.52 ± 4.02			48.20 ± 3.87		
Parametrial invasion						
No	43.68 ± 3.01	−22.467–7.181	0.877	46.18 ± 2.43	−21.708–4.791	0.295
Unilateral	51.32 ± 4.13			54.63 ± 3.15		
Bilateral	40.25 ± 2.58			44.27 ± 4.21		
Clinical lymph node involvement						
cN0	48.80 ± 2.87	1.140–25.259	0.032	51.72 ± 2.55	1.283–22.960	0.029
cN1	35.60 ± 5.96			39.60 ± 5.55		
FIGO stage						
II	45.78 ± 3.51	−10.341–12.072	0.878	52.57 ± 2.77	0.286–19.813	0.044
III	44.91 ± 4.32			42.52 ± 4.28		
Hemoglobin levels at diagnosis (g/dL)						
≤113	44.14 ± 6.41	−11.144–14.545	0.792	47.08 ± 6.16	−9.923–13.816	0.743
>113	45.84 ± 2.97			49.02 ± 2.64		
Platelets at diagnosis (g/dL)						
≤320	36.50 ± 8.30	−25.057–3.521	0.136	42.60 ± 7.69	−20.208–5.738	0.269
>320	47.27 ± 2.75			49.83 ± 2.49		
Response						
CR	48.09 ± 3.47	−4.490–17.499	0.241	52.11 ± 2.89	−0.9463–18.740	0.075
Non-CR	41.58 ± 4.26			43.22 ± 4.15		
NLR						
≥2.0	46.94 ± 3.49	−9.711–14.331	0.701	43.06 ± 3.61	−21.842–0.356	0.043
<2.0	44.63 ± 4.96			54.16 ± 3.15		

PFS, progression-free survival; OS, overall survival; LACSCC, locally advanced cervical squamous cell carcinomas; FIGO, International Federation of Gynecology and Obstetrics; CR, complete response; NLR, neutrophil-lymphocyte ratio.

**Table 3:** Multivariate survival analysis of OS in patients with LACSCC.

Clinicopathologic characteristics	*B*	SE	Wald	*P* value	HR	95% CI
Lymph node metastasis	0.626	0.516	1.475	0.225	1.870	0.681–5.139
FIGO stage	0.188	0.495	0.144	0.704	1.207	0.457–3.185
NLR	−1.316	0.631	4.350	0.037	0.268	0.078–0.924

OS, overall survival; LACSCC, locally advanced cervical squamous cell carcinomas; FIGO, International Federation of Gynecology and Obstetrics; NLR, neutrophil-lymphocyte ratio.

**Table 4:** Association between RNG and clinicopathologic characteristics of LACSCC patients with neutropenia.

Clinicopathologic characteristics	RNG, *n* (%)		*P* value
	≥3.01	<3.01	
Age			
≤50 years	11 (52.4)	10 (47.6)	0.890
>50 years	7 (50.0)	7 (50.0)	
Histologic grade			
Well and moderately differentiated	7 (50.0)	7 (50.0)	0.890
Poorly differentiated	11 (52.4)	10 (47.6)	
Tumor size			
≤4 cm	5 (55.6)	4 (44.4)	0.921
>4 cm	13 (50.0)	13 (50.0)	
Parametrial invasion			
No	6 (42.9)	8 (57.1)	0.462
Unilateral	9 (64.3)	5 (35.7)	
Bilateral	3 (42.9)	4 (57.1)	
Clinical lymph node involvement			
cN0	7 (33.3)	14 (66.7)	0.023
cN1	11 (78.6)	3 (21.4)	
FIGO stage			
II	9 (52.9)	8 (47.1)	0.862
III	9 (50.0)	9 (50.0)	
Hemoglobin levels at diagnosis (g/dL)			
≤113	4 (44.4)	5 (55.6)	0.921
>113	14 (53.8)	12 (46.2)	
Platelets at diagnosis (g/dL)			
≤320	3 (75.0)	1 (25.0)	0.638
>320	15 (48.3)	16 (51.7)	
Response			
CR	7 (36.8)	12 (63.2)	0.060
Non-CR	11 (68.8)	5 (31.2)	

RNG, responses of neutrophil to granulocyte colony-stimulating factors; LACSCC, locally advanced cervical squamous cell carcinomas; FIGO, International Federation of Gynecology and Obstetrics; CR, complete response.

**Table 5:** Univariate survival analysis of OS for LACSCC patients with neutropenia.

Clinicopathologic characteristics	OS		*P* value
	Mean ± SD (months)	95% CI	
Age			
≤50 years	45.21 ± 4.54	−21.451–3.589	0.155
>50 years	54.14 ± 3.60		
Histologic grade			
Well and moderately differentiated	51.64 ± 4.19	−8.242–17.422	0.471
Poorly differentiated	47.05 ± 4.42		
Tumor size			
≤4 cm	47.33 ± 3.90	−20.303–8.075	0.386
>4 cm	53.44 ± 4.40		
Parametrial invasion			
No	45.15 ± 3.19	−27.924–13.195	0.420
Unilateral	54.07 ± 2.56		
Bilateral	46.71 ± 4.37		
Clinical lymph node involvement			
cN0	54.50 ± 2.96	−26.016–1.912	0.025
cN1	40.54 ± 5.76		
FIGO stage			
II	50.71 ± 3.88	−16.257–9.215	0.057
III	47.19 ± 4.93		
Hemoglobin levels at diagnosis (g/dL)			
≤113	47.70 ± 3.95	−9.524–18.138	0.530
>113	52.00 ± 4.73		
Platelets at diagnosis (g/dL)			
≤320	49.24 ± 3.27	−17.599–21.580	0.837
>320	47.25 ± 10.49		
Response			
CR	44.80 ± 5.15	−20.231–4.832	0.219
Non-CR	52.50 ± 3.60		
RNG			
≥3.01	40.06 ± 4.80	7.571–29.312	0.002
<3.01	58.50 ± 2.00		

OS, overall survival; LACSCC, locally advanced cervical squamous cell carcinomas; FIGO, International Federation of Gynecology and Obstetrics; CR, complete response; RNG, responses of neutrophil to granulocyte colony-stimulating factors.

**Table 6:** Multivariate survival analysis of OS for LACSCC patients with neutropenia.

Clinicopathologic characteristics	*B*	SE	Wald	*P* value	HR	95% CI
Lymph node metastasis	0.668	0.696	0.921	0.337	1.951	0.498–7.635
FIGO stage	0.326	0.753	0.188	0.665	1.385	0.317–6.056
RNG	1.833	0.956	3.678	0.055	6.252	0.961–40.687

OS, overall survival; LACSCC, locally advanced cervical squamous cell carcinomas; FIGO, International Federation of Gynecology and Obstetrics; RNG, responses of neutrophil to granulocyte colony-stimulating factors.

**Table 7:** The summary of NLR studies in cervical cancer.

Patient number	Median age (range, years)	FIGO stage	Histology	Treatment	Median NLR/cut-off value of NLR	Association of NLR with clinicopathologic characteristics	Prognostic factor determined in multivariable analysis	Reference
1061	50 (21–85)	IB1-IVA	SCC, AC, ASC	Surgery + RT or CRT	1.9/1.9	Age, stage, treatment modality	NLR	[9]

111	42 (21–68)	IB2-IIB	SCC, non-SCC	NCT + surgery	2.4/2.5	FIGO stage	LN metastasis, lymphovascular space involvement	[10]

460	44 (24–78)	I-II	SCC, AC	Surgery + RT	2.213/2.213	Depth of stromal infiltration, LN metastasis	NLR, FIGO stage and LN metastasis	[11]

56	65.1 (35–89)	IB1-IV	SCC	RT or CRT	2.4/2.5	FIGO stage, mean SCC value, tumor size, LN metastasis, CR	NLR	[12]

60	53 (36–80)	II-III	SCC	CRT	2.4/2.0	Age, baseline platelet count	NLR	Current study

NLR, neutrophil-lymphocyte ratio; FIGO, International Federation of Gynecology and Obstetrics; SCC, squamous cell carcinoma; AC, adenocarcinoma; ASC, adenosquamous cell carcinoma; RT, radiotherapy; CRT, chemoradiotherapy; NCT, neoadjuvant chemotherapy; LN, lymph node; CR, complete response.
